# Spontaneous Left Anterior Descending Artery Dissection in a Middle-Aged Woman with Vitamin B12 Deficiency Treated with Coronary Artery Bypass Grafting

**DOI:** 10.7759/cureus.2902

**Published:** 2018-07-01

**Authors:** Quratulain Fatima Masood, Ali Asad, Syed Maaz Tariq, Saad Javaid, Muhammad H Khalil, Fiaz Hussain

**Affiliations:** 1 Surgery, National University of Science and Technology, Rawalpindi, PAK; 2 Pediatric Surgery, University of Health Sciences, Lahore, PAK; 3 Medicine, Jinnah Sindh Medical University (SMC), Karachi, PAK; 4 Medicine, Nishtar Medical University/Nishtar Hospital, Multan, PAK; 5 Internal Medicine, University of Health Sciences, Detroit, USA; 6 Medicine, Nishtar Medical University, Multan, PAK

**Keywords:** coronary artery, dissection, lad

## Abstract

Spontaneous coronary artery dissection (SCAD) is one of the rarest causes of acute coronary syndromes, which include myocardial infarction (MI), stable and unstable angina, cardiogenic shock, and sudden death. The course of the disease, its etiology, prevalence, prognosis, and treatment remain ill-defined. Adding to the complexity is the fact that patients may lack typical risk factors for coronary heart disease. Herein, we report a case of a 42-year-old woman with vitamin B12 deficiency, who presented with chest pain; electrocardiography (ECG) findings were consistent with the acute anterior wall MI. Cardiac catheterization was done, which showed a very large left anterior descending (LAD) artery dissection.

## Introduction

Spontaneous coronary artery dissection (SCAD) is an extremely rare cause of acute coronary syndromes, which include myocardial infarction (MI), stable and unstable angina, cardiogenic shock, and sudden death [1]. Diagnosis can be made through clinical picture, cardiac catheterization and more frequently by autopsy [2]. The pathogenesis and treatment remain unclear. The patients may lack typical risk factors for coronary heart disease as well. Up to a third of all reported cases have occurred during the third trimester of pregnancy or early in the postpartum period. The mechanism is not completely understood but the hemodynamic changes in pregnancy and hormone-mediated alterations are said to be related to SCAD during pregnancy. Pregnancy-induced changes in arterial walls such as fragmentation of reticulin fibers may also result in dissection [3].

## Case presentation

**Figure 1:**
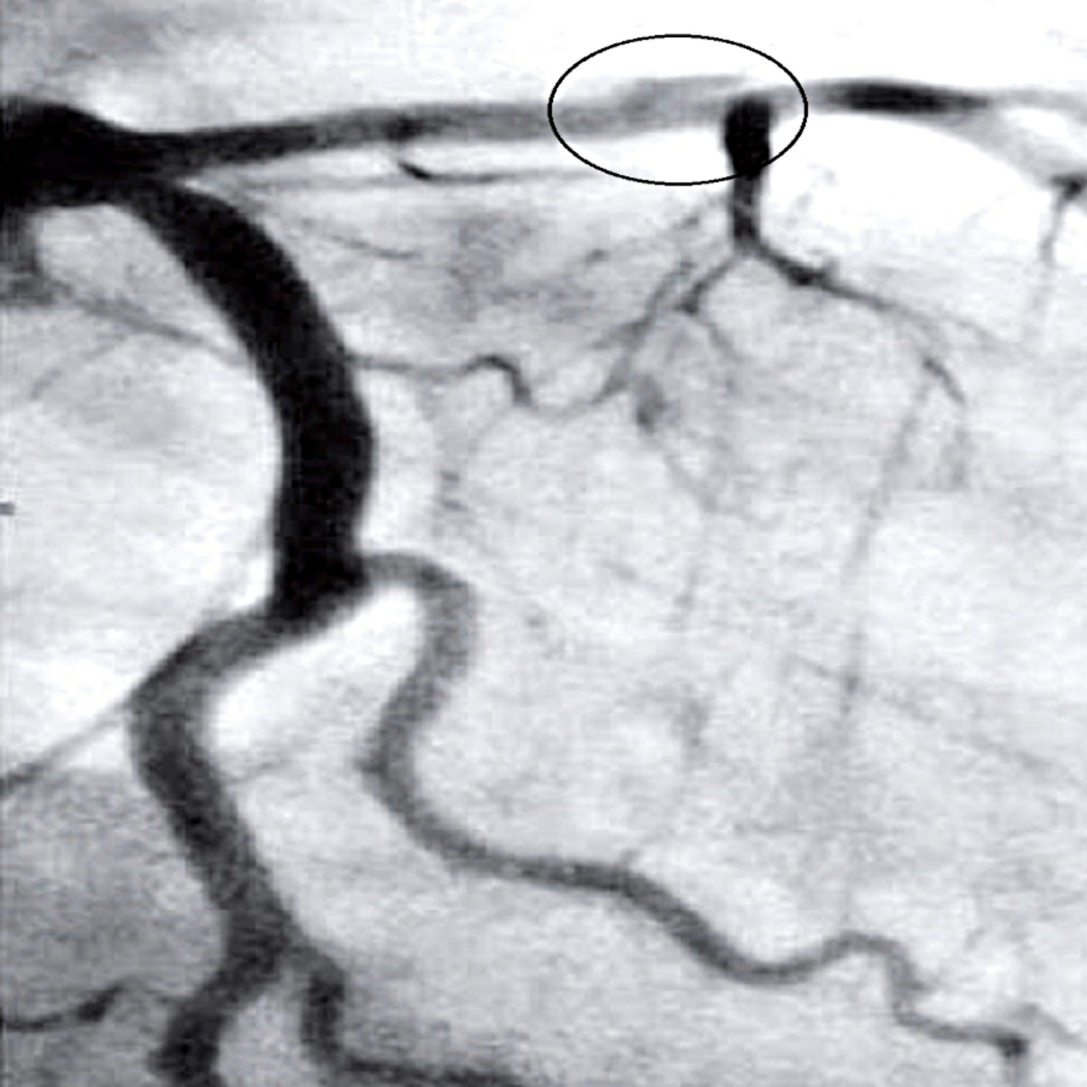
Angiograph showing left anterior descending (LAD) artery dissection.

A 42-year-old nonpregnant, housewife with no known comorbidities, presented to the emergency room (ER) with chest pain for 15 minutes where electrocardiography (ECG) was done and it showed acute onset anterior wall ST-segment elevation MI. She was kept in coronary care unit (CCU) under observation for two days; no thrombolysis was done because the patient had a history of bleeding peptic ulcer and, therefore, was started on aspirin, clopidogrel, and simvastatin where her ST-segment elevation settled but again started to rise. Hence, angiography was done that showed dissection in left anterior descending (LAD) artery (Figure 1).

The patient had no family history of sudden cardiac death, coronary heart disease, or coronary artery dissection; past medical, surgical, and psychosocial histories were unremarkable. Physical examination was unremarkable. Chest radiography was normal. Cardiac enzymes were normal. The echocardiogram was normal. Due to recurrent chest pain, emergent off-pump CABG was performed, and the left internal mammary artery was anastomosed to the LAD distally. She had an uneventful recovery and during 18 months follow-up period, the patient was free of chest pain and her ECG became normal.

## Discussion

Spontaneous coronary artery dissection has been reported with multiple factors including the peripartum period [4-5], postpartum period [6-7], oral contraceptives use [8], and connective tissue disorders including Ehlers-Danlos syndrome [9], systemic lupus erythematosus [10], neurofibromatosis [11], sexual intercourse [12], and bleomycin-etoposide-cisplatin therapy for testicular cancer [13]. It occurs most commonly in females and the LAD is the vessel affected most often [14]. There have been no previous case reports describing any link between SCAD and vitamin B12 deficiency, which would have been treated successfully with off-pump CABG. Elevated homocysteine level due to vitamin B12 deficiency has been linked with spontaneous cervical artery dissection [15], so we can think that there may be a linkage between SCAD and vitamin B12 deficiency because apart from that the patient did not have any other cardiac risk factors. The optimum management of SCAD is not well defined. Possible treatment options include medical therapy (aspirin, heparin, beta blockers, statins, and nitrates) or invasive therapies like CABG, angioplasty and stenting and even cardiac transplantation [16]. Thrombolysis is a therapeutic option [17], but there are cases reported in the literature where thrombolysis resulted in further increase of the dissection [18]. Bypass surgery has been used in numerous cases especially for LAD dissection or when the initial therapies were unsatisfactory [19]. Surgical therapy was pursued after considering the following factors: length of the dissection and presence of symptoms at the time of angiography. In retrospect, it appears to have been a good decision as the patient made a fully uneventful recovery without any complications at all. For perfectly healthy young patients presenting with LAD dissection and having any contraindication for thrombolysis like a history of bleeding peptic ulcer in our case, CABG can be employed for treatment. However, coronary artery stenting could be a good option for SCAD especially in monotruncular lesion [20]. Prognosis of the SCAD is better if the patient is able to go through the initial event. Hence, we conclude that early intervention with CABG in healthy patients presenting with LAD dissection leads to the desired outcome and less need for further interventions.

## Conclusions

This case is rare in that SCAD occurred in a young woman who was nonpregnant and without any risk factors associated with SCAD, who had low vitamin B12 levels (140 pg/dl) which causes increased homocysteine levels that is further linked with spontaneous carotid artery dissection. In a patient like this where there were no cardiac risk factors, elevated homocysteine due to B12 deficiency can be linked to SCAD. But as there are no cases reported like this, more research and population studies are required to further know about it. Family history, psychosocial history, past medical history, past surgical history, and physical examination were unremarkable; cardiac enzymes, radiography, and echocardiogram were perfectly normal. We propose that vitamin B12 deficiency could be associated with an increased risk for SCAD in young healthy women without any comorbids.

## References

[1] Leone F, Macchiusi A, Ricci R, Cerquetani E, Reynaud M (2004). Acute myocardial infarction from spontaneous coronary artery dissection a case report and review of the literature. Cardiol Rev.

[2] DeMaio Jr SJ, Kinsella SH, Silverman ME (1989). Clinical course and long-term prognosis of spontaneous coronary artery dissection. Am J Cardiol.

[3] Havakuk O, Goland S, Mehra A, Elkayam U (2017). Pregnancy and the risk of spontaneous coronary artery dissection: an analysis of 120 contemporary cases. Circ Cardiovasc Interv.

[4] Hammond AS, Bailey PL (2006). Acute spontaneous coronary artery dissection in the peripartum period. J Cardiothorac Vasc Anesth.

[5] Engelman DT, Thayer J, Derossi J, Scheinerman J, Brown L (1993). Pregnancy related coronary artery dissection: a case report and collective review. Conn Med.

[6] Halmai L, Sepp R, Thury A, Gavallér H, Ungi I, Rudas L (2008). Coronary artery dissection in the postpartum period-a case study. Orv Hetil.

[7] Kalra N, Greenblatt J, Ahmed S (2008). Postpartum spontaneous coronary artery dissection (SCAD) managed conservatively. Int J Cardiol.

[8] Heefner WA (1973). Dissecting hematoma of the coronary artery: a possible complication of oral contraceptive therapy. J Am Med Assoc.

[9] Catanese V, Venot P, Lemesle F, Delille F, Runge I, Kuchly B (1995). Myocardial infarction by spontaneous dissection of coronary arteries in a subject with type IV Ehlers-Danlos syndrome. Presse Med.

[10] Sharma AK, Farb A, Maniar P (2003). Spontaneous coronary artery dissection in a patient with systemic lupus erythematosis. Hawaii Med J.

[11] Giugliano GR, Sethi PS (2009). Spontaneous left anterior descending coronary artery dissection in a patient with neurofibromatosis. J Invasive Cardiol.

[12] Schifferdecker B, Pacifico L, Ramsaran EK, Folland ED, Spodick DH, Weiner BH (2004). Spontaneous coronary artery dissection associated with sexual intercourse. Am J Cardiol.

[13] Ghosh N, Chow CM, Korley V, Chisholm R (2008). An unusual case of chronic coronary artery dissection: did cisplatin play a role?. Can J Cardiol.

[14] Jorgensen MB, Aharonian V, Mansukhani P, Mahrer PR (1994). Spontaneous coronary dissection: a cluster of cases with this rare finding. Am Heart J.

[15] Arauz A, Hoyos L, Cantu C (2007). Mild hyperhomocysteinemia and low folate concentrations as risk factors for cervical arterial dissection. Cerebrovasc Dis.

[16] Eddinger J, Dietz WA (2005). Recurrent spontaneous coronary artery dissection. Catheter Cardiovasc Interv.

[17] Satler LF, Levine S, Kent KM (1984). Aortic dissection masquerading as acute myocardial infarction: implication for thrombolytic therapy without cardiac catheterization. Am J Cardiol.

[18] Buys EM, Suttorp MJ, Morshuis WJ, Plokker HW (1994). Extension of a spontaneous coronary artery dissection due to thrombolytic therapy. Cathet Cardiovasc Diagn.

[19] Klutstein MW, Tzivoni D, Bitran D, Mendzelevski B, Ilan M, Almagor Y (1997). Treatment of spontaneous coronary artery dissection: report of three cases. Cathet Cardiovasc Diagn.

[20] Gonzalez JI, Hill JA, Conti CR (1989). Spontaneous coronary artery dissection treated with percutaneous transluminal angioplasty. Am J Cardiol.

